# Tumor‐Targeted Injectable Double‐Network Hydrogel for Prevention of Breast Cancer Recurrence and Wound Infection via Synergistic Photothermal and Brachytherapy

**DOI:** 10.1002/advs.202200681

**Published:** 2022-06-25

**Authors:** Yuanhao Wu, Yuan Yao, Jiamin Zhang, Han Gui, Jinjian Liu, Jianfeng Liu

**Affiliations:** ^1^ Key Laboratory of Radiopharmacokinetics for Innovative Drugs Chinese Academy of Medical Sciences and Institute of Radiation Medicine Chinese Academy of Medical Sciences & Peking Union Medical College Tianjin 300192 China; ^2^ Lab of Functional and Biomedical Nanomaterials College of Materials Science and Engineering Qingdao University of Science and Technology Qingdao 266042 China

**Keywords:** brachytherapy, breast cancer, double‐network hydrogel, photothermal therapy, wound infection

## Abstract

The high locoregional recurrence rate and potential wound infection in breast cancer after surgery pose enormous risks to patient survival. In this study, a polyethylene glycol acrylate (PEGDA)‐alginate double‐network nanocomposite hydrogel (GPA) embedded with ^125^I‐labeled RGDY peptide‐modified gold nanorods (^125^I‐GNR‐RGDY) is fabricated. The double‐network hydrogel is formed by injection of GPA precursor solutions into the cavity of resected cancerous breasts of mice where gelation occurred rapidly. The enhanced temperature‐induced PEGDA polymerization driven by near‐infrared light irradiation, and then, the second polymer network is crosslinked between alginate and endogenous Ca^2+^ around the tumor. The double‐network hydrogel possesses a dense polymer network and tightly fixes ^125^I‐GNR‐RGDY, which exhibit superior persistent photothermal and radioactive effects. Hyperthermia induced by photothermal therapy can inhibit self‐repair of damaged DNA and promote blood circulation to improve the hypoxic microenvironment, which can synergistically enhance the therapeutic efficacy of brachytherapy and simultaneously eliminate pathogenic bacteria. Notably, this nanocomposite hydrogel facilitates antibacterial activity to prevent potential wound infection and is tracked by single‐photon emission computerized tomography imaging owing to isotope labeling of loaded ^125^I‐GNR‐RGDY. The combination of photothermal therapy and brachytherapy has enabled the possibility of proposing a novel postoperative adjuvant strategy for preventing tumor recurrence and wound infection.

## Graphical abstract

An injectable double‐network nanocomposite hydrogel is engineered by near‐infrared (NIR)‐induced polymerization of polyethylene glycol acrylate and ionic crosslink between alginate and endogenous Ca^2+^ after injection of precursor solutions into the cavity of resected breasts. The double‐network hydrogel efficiently prevents postoperative tumor recurrence and wound infection via synergistic NIR‐stimuli photothermal and brachytherapy.

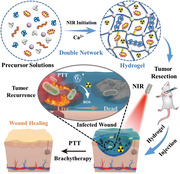

## 1 Introduction

Breast cancer, the most commonly occurring cancer among women worldwide, is diagnosed in 12% of women over their lifetime and accounted for 30% of cancer cases among women in 2020.^[^ ^1^, ^2^ ^]^ Clinically, surgical resection remains the primary form of intervention for the comprehensive treatment of breast cancer, and more than 90% of early breast cancer patients undergo surgical treatment, including mastectomy and breast‐conserving surgery.^[^ ^3^ ^]^ However, the high local recurrence rate of breast cancer remains a fatal problem in the clinic; thus, almost all surgery patients are required to undergo lengthy recurrence therapy.^[^ ^4^ ^]^ In addition, the potential risk of surgical infection and low immunity of patients after tumor surgery increase the chances of bacterial infections that may pose serious complications and a threat to their survival.^[^ ^5^, ^6^ ^]^ Traditional cancer treatments such as chemotherapy, radiotherapy (RT), and immunotherapy are rather restricted in addressing these multiple requirements in complicated postoperative tumor therapy. Therefore, there is an urgent need to develop an intelligent adjuvant therapeutic strategy to simultaneously prevent tumor recurrence and potential wound infections after tumor resection.

Adjuvant RT after breast‐conserving surgery is the standard of care for the local management of tumor recurrence and can remarkably reduce the risk of locoregional recurrence and enhance breast cancer‐specific survival.^[^ ^7^ ^]^ However, external beam radiotherapy (EBRT) is potentially associated with more toxicity to the normal tissue, and a few patients with breast cancer may experience local recurrence even after undergoing RT after surgery.^[^ ^8^ ^]^ Brachytherapy, one of the RT modalities, has been widely applied in the clinic for tumor treatment owing to its prominent advantages of precisely localized radiation with short‐range and highly efficient antitumor effects as well as lower toxicity to normal tissues compared with EBRT. ^125^I, with its half‐life of 60.14 days, is a therapeutic radioisotope emitting *γ* particles (0.03548 MeV), which have been widely applied in brachytherapy and isotope imaging in clinic.^[^ ^9^ ^]^ Radioactive ^125^I seed implantation has been proved to be an efficient way to sustainable killing for tumor cells without any significant injury to the neighboring cells, which is particularly useful in controlling the local tumor growth.^[^ ^10^ ^]^ Nevertheless, the migration of radioisotopes after permanent implantation is also a critical reason for brachytherapy complications.^[^ ^11^ ^]^ Moreover, the intrinsic self‐healing capacity of DNA and local tumor hypoxic microenvironment are capable of reducing the tumor elimination capability of RT and may even result in tumor deterioration through random duplication and repair of the damaged DNA.^[^ ^12^, ^13^, ^14^ ^]^ Therefore, traditional therapy in clinics, such as RT, is not sufficient or effective in inhibiting breast cancer recurrence. Recent studies have demonstrated that hyperthermia induced by photothermal therapy (PTT) can prevent DNA self‐repair and promote blood circulation to improve the hypoxic microenvironment, which can synergistically enhance the therapeutic efficacy of RT for cancer treatment.^[^ ^15^ ^]^ However, the limited penetration depth of near‐infrared (NIR) light may act as a barrier for photothermal activity and consequently restrict its clinical applications. Herein, the combinatorial treatment modality involving PTT and RT was able to compensate for the penetration depth of NIR and synergistically improve the therapeutic efficacy for tumor therapy.

The emergence of nanotechnology and tremendous advances in stimuli‐responsive hydrogels have enabled the possibility of proposing new strategy for surgical adjuvant combination therapy.^[^ ^16^ ^]^ It has been noticed that the multifunctional nanocomposite hydrogel combined with photothermal therapy is developed to be an effective approach for synergistic tumor therapy and antibacterial treatment.^[^ ^17^ ^]^ To locally inhibit tumor recurrence and wound infection, implantable hydrogels are favorable for the precise implementation of photothermal ablation and continuous elimination of pathogenic bacteria because of their persistent therapeutic effects around residual tumor sites.^[^ ^18^, ^19^, ^20^, ^21^, ^22^ ^]^ Injectable hydrogels, constructed through dynamic crosslinks, have been widely applied for tumor therapy because of their minimally invasive and tumor‐targeting properties, which can be transformed from sol to gel state in situ and will aid in realizing local precision for cancer therapy.^[^ ^23^, ^24^, ^25^, ^26^ ^]^ In particular, this class of hydrogels could be triggered by the tumor microenvironment (such as pH, physical environment, and cellular metabolism), leading to a specific application to tumor cells with precise effects of PTT and brachytherapy.^[^ ^27^ ^]^ For instance, alginate can interact with endogenous Ca^2+^ to transform into a hydrogel after injection into the tumor and could efficiently load a radioisotope‐labeled agent for tumor RT.^[^ ^28^ ^]^ Despite the encouraging results of radioisotope therapy against breast cancer, the uncontrollable release and weak stability of radioisotopes are not favorable for long‐term inhibition of breast cancer recurrence.

For inhibition of locoregional recurrence and potential wound infection, an ideal hydrogel for the postoperative treatment of breast cancer is supposed to possess the following features: tumor microenvironment and thermoresponsive‐triggered gelation property, rapid gelation time, physiological stability and biosafety, long‐term self‐imaging capacity (e.g., autoradiograph), and excellent therapeutic efficacy in terms of both antitumor and antibacterial activities.

A recent study demonstrated that radiolabeled nanoparticles can simultaneously act as photothermal and brachytherapy agents for the combination of PTT and RT.^[^ ^27^ ^]^ However, the weak retention and uncontrollable migration of radiolabeled nanoparticles pose a significant challenge for continuous and effective tumor therapy. The double‐network (DN) strategy can enhance the mechanical properties and stability of hydrogels through a tight polymer network and superior load‐bearing properties, owing to the energy dissipation mechanism of the specific combination of two networks with contrasting structures.^[^ ^29^ ^]^ Therefore, radiolabeled nanoparticle‐loaded DN hydrogels may pave the way for future applications of the combination of PTT and brachytherapy for postoperative cancer treatment.

**Scheme 1:**
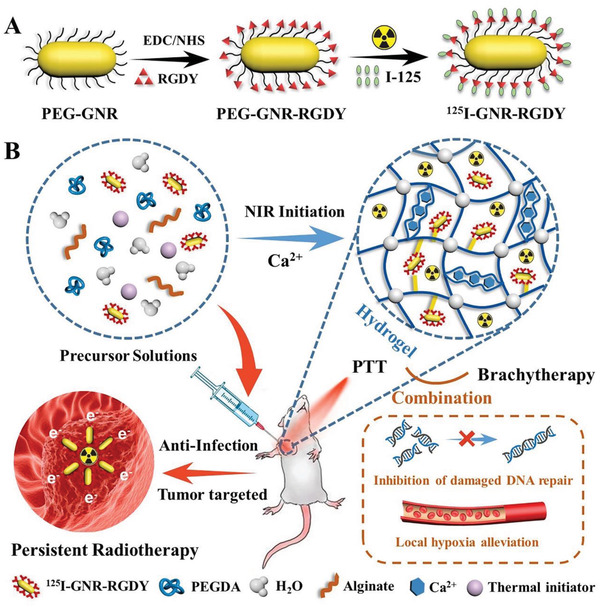
Schematic illustration of the fabrication of A) ^125^I‐GNR‐RGDY and B) nanocomposite double‐network GPA hydrogel and their theranostic application for inhibition of postoperative breast cancer recurrence and wound infection through synergistic brachytherapy and photothermal therapy.

In this study, we designed and fabricated a polyethylene glycol acrylate‐alginate DN nanocomposite hydrogel (GPA) bearing ^125^I‐labeled RGDY‐modified gold nanorods (^125^I‐GNR‐RGDY) for the combined postoperative prevention of local recurrence and wound infection in breast cancer through synergetic PTT and brachytherapy. We hypothesized that ^125^I‐GNR‐RGDY entrapped in the hydrogel could thermally initiate the polymerization of polyethylene glycol acrylate (PEGDA) under NIR irradiation owing to the photothermal conversion of GNR, followed by the formation of a second crosslink between alginate and endogenous Ca^2+^ around the tumor to produce the DN hydrogel. The tight fixation of ^125^I‐GNR‐RGDY arising from the denser polymer network of the DN hydrogel would maintain excellent photothermal activity and thereby contribute to the effective prevention of local recurrence of breast cancer by synergetic NIR‐stimulated PTT and brachytherapy. Furthermore, ^125^I‐GNR‐RGDY possesses superior affinity for tumor tissues and specifically accumulates in residual tumor cells owing to the tumor‐targeting efficiency of the RGD peptide, which led to an accurate therapeutic effect of PTT and brachytherapy. Moreover, as a safe external stimulus, NIR‐remoted PTT could inhibit self‐repair of the damaged DNA by brachytherapy and overcome the hypoxia‐associated RT resistance by enhancing blood circulation, which was favorable for the combination and complementation of PTT and brachytherapy for cancer treatment. Notably, PTT‐induced local hyperpyrexia in the environment could simultaneously eliminate potential pathogenic bacteria during postoperative cancer therapy, leading to persistent prevention of bacterial wound infection. In addition, the nanocomposite hydrogel could be tracked in vivo by isotope and single‐photon emission computerized tomography (SPECT) imaging because of the radiolabeling of GNR. Fabrication of the tumor‐targeted nanocomposite hydrogel (^125^I‐GPA) and synergistic treatment with PTT and brachytherapy for breast cancer recurrence and wound infection are depicted in **Scheme** **1**.

## 2 Results and Discussion

### 2.1 Characterization of ^125^I‐GNR‐RGDY

**Figure 1:**
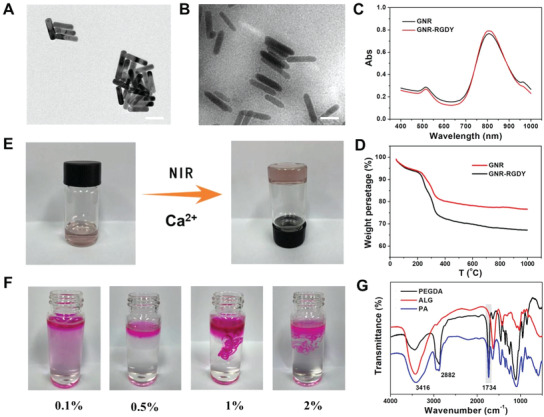
TEM images of A) GNR and B) GNR‐RGDY. C) UV–vis absorption spectra of GNR and GNR‐RGDY. Scale bar: 50 nm. D) TGA curves recorded for GNR (black) and GNR‐RGDY (red). The content of RGDY in was calculated by the weight loss from 200 to 1000 °C. E) Photographs showing the formation of GPA hydrogel. F) Photographs showing the gelation performance of ALG at different concentrations (0.1, 0.5, 1, and 2 wt%) after injection into Ca^2+^ containing PBS. G) FTIR spectra of PEGDA, ALG, and PA hydrogels.

In this study, we aimed to design a tumor microenvironment‐responsive hydrogel for synergistic inhibition of postoperative breast cancer recurrence and drug‐resistant infection. Considering the prominent tumor‐targeting efficacy and photothermal effect of RGD peptide‐modified GNR as well as the isotope imaging following radiolabeling in vivo, we synthesized an iodine‐labeled RGDY‐conjugated gold nanorod (^125^I‐GNR‐RGDY). GNR‐RGDY was first synthesized by exploiting the carbodiimide chemistry between the amino group of the RGDY peptide and the carboxyl group of PEG‐modified GNR, followed by ^125^I radiolabeling of tyrosine onto the nanoparticles, as previously reported.^[^ ^30^ ^]^ According to the standard chloramine‐T method, GNR‐RGDY was labeled with ^125^I at a high radiolabeling rate of 90.8% and radiochemical purity of 99.7% (Figure S1, Supporting Information). Notably, ^125^I‐GNR‐RGDY was only applied in cell and animal experiments because of the restriction of isotope application; therefore, GNR‐RGDY was used for the characterization of nanoparticles. The variations in morphology and size between GNR and GNR‐RGDY were observed by transmission electron microscopy (TEM). As shown in **Figure** **1**A, GNR demonstrated a claviform shape with an average particle diameter of 79.3 ± 3.1 nm and a length‐to‐diameter ratio of 4:3, which was in the same range as its hydrodynamic diameter, 92.3 ± 10.0 nm, calculated by dynamic light scattering (DLS) (Figures S2 and S3, Supporting Information). After conjugation of RGDY, the particle diameter increased to 102.2 ± 5.9 nm (Figure 1B), which was in the same range as the hydrodynamic diameter, 136.7 ± 15.2 nm, as calculated by DLS.

To explore the differences in adsorption after conjugation of RGDY, the UV–vis absorption spectra of GNR and GNR‐RGDY were determined. As shown in Figure 1C, the two absorption peaks of GNR and GNR‐RGDY almost overlapped around 520 and 810 nm, indicating an absence of influence of the photothermal property after the modification of RGDY into GNR. Notably, the wavelength of the characteristic absorbance peak of GNR‐RGDY, which was close to the light source wavelength (808 nm), may lead to more potent photothermal activity. Furthermore, the amount of RGDY conjugated onto GNR was measured by thermogravimetric analysis (TGA) based on the weight loss of GNR‐RGDY as compared with that of GNR. The weight loss of GNR between 25 and 1000 °C was ≈23%, corresponding to the decomposition of modified PEG on GNR. The weight loss increased to ≈33% for GNR‐RGDY, indicating that the weight percentage of RGDY was ≈10% (Figure 1D).

### 2.2 Characterization of Nanocomposite Hydrogels

The nanocomposite hydrogel was composed of GNR‐RGDY and PEGDA–alginate (ALG) DN hydrogel (GPA). PEGDA and ALG monomers were added to GNR or GNR‐RGDY precursor solution. Thermal induction of PEGDA polymerization led to formation of the first polymer network under conditions involving NIR irradiation based on the photothermal property of GNR. This was followed by immersion into Ca^2+^ solutions to construct a DN hydrogel because of strong interactions between ALG and Ca^2+^ (Figure 1E). To confirm the feasibility of the secondary crosslinking of ALG in the tumor microenvironment, the ALG concentration‐dependent gel formation process was examined at physiological concentrations of Ca^2+^ (1.8 × 10^−3^ m). GPA precursor solutions with various concentrations were injected into Ca^2+^‐containing phosphate‐buffered saline (PBS) to test the gelation performance. As depicted in Figure 1F, the fluid‐like behavior of ALG was observed at a concentration lower than 0.5 wt%, while the mixture at increasing concentrations (≥1 wt%) was able to rapidly transform into gels in the presence of Ca^2+^. This result further confirmed the potential feasibility of DN hydrogel formation around tumor sites in vivo.

Structural characterization of the DN hydrogel was performed by Fourier transform infrared (FTIR) spectroscopy. As shown in Figure 1G, the absorption peak of PA at 3416 cm^−1^ was assigned to the OH symmetrical stretching vibration, consistent with that of ALG. Furthermore, typical adsorption bands of PEGDA at 2882 and 1734 cm^−1^ were observed in the FTIR spectrum of PA, which were assigned to the symmetrical stretching vibration of ‐CH_2_‐ and C=O, respectively. This result confirmed the successful synthesis of the PA hydrogel. After immersion into deionized water for 7 days, the equilibrium water contents of PA and GPA hydrogels were determined to be 85.2 and 83.8 wt%, respectively (Figure S4, Supporting Information).

The differences in the crosslinking density and hydrogel network between PEGDA and PA were evaluated by scanning electron microscopy (SEM). As shown in Figure S5 (Supporting Information), the PA hydrogel displayed tight and nanofibrous intertwining structures as compared with the loosened network of PEGDA, which was attributed to the additional crosslinking of the DN between ALG and Ca^2+^. Importantly, the tight fixation of GNR in the networks prevented leakage of the hydrogel and thereby enhanced the photothermal activity of the GNR‐loaded hydrogel (discussed later). According to previous study, PEGDA can be injected into tumors and quickly converted to a hydrogel for fixation of CuS nanoparticles via remotely controlled polymerization triggered by the NIR laser.^[^ ^27^ ^]^ However, the weak mechanical property and instability of PEGDA hydrogel lead to undesirable breakage and then accelerate the release of the loaded nanoparticles, which may influence the photothermal effects in long‐term postoperative tumor therapy. Thus, the long‐term stability of the hydrogel immersed in water and PBS was tested to verify the formation of the DN structure. As depicted in Figure S6 (Supporting Information), we observed an obvious weight loss of PEG in the first 3 days, and the degradation rate increased to 25.3% and 25% in water and PBS on day 28, respectively. On the contrary, the degradation rate of PA hydrogel was maintained below 10% after immersion in water and PBS for 28 days. These results further confirm the superior stability of the PA hydrogel in a physiological environment, owing to the dense structure and better mechanical properties of the DN hydrogel.

### 2.3 Photothermal Effects of GPA Hydrogel

**Figure 2:**
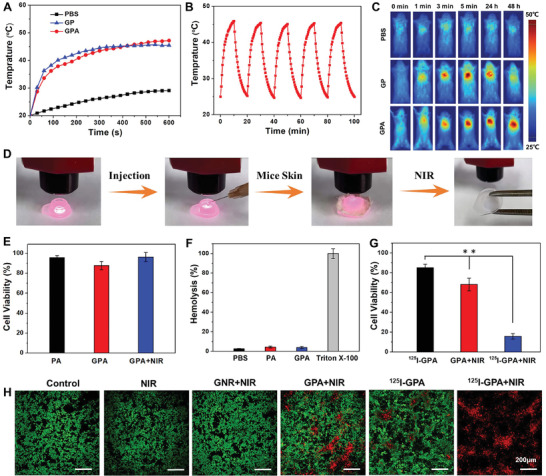
A) Photothermal activity of PBS, GP, and GPA hydrogels under NIR irradiation (808 nm, 0.5 W cm^−2^) in 10 min. B) Photothermal conversion cycling test of GPA hydrogel under NIR irradiation (808 nm, 0.5 W cm^−2^). C) Infrared thermal images of mice injected with PBS, GNR, and GPA hydrogel under NIR irradiation (0.5 mW cm^−2^) through an infrared thermal camera. After implantation of GPA hydrogel for 24 and 48 h, the temperature at implanting position was measured after irradiation for 10 min. D) Schematic photograph of NIR‐simulated gelation process through mice skin. After injection of precursor solutions of GPA, the rat skin was covered onto the solutions, followed by the NIR irradiation for 1 min to form the hydrogel. E) Cell viability of L929 cells treated with PA and GPA hydrogels, as well as GPA hydrogel under laser irradiation (*n* = 5). F) Hemolytic activity of PA and GPA hydrogels with erythrocyte stock in Tris buffer solution (*n* = 5). PBS and Triton X‐100 served as negative and positive control, respectively. G) Cell viability assay of 4T1 cells treated with ^125^I‐GPA and GPA hydrogels with or without NIR irradiation (*n* = 5). H) Live/dead staining results of 4T1 cells after incubation with PBS, GNR, GPA, and ^125^I‐GPA hydrogels with or without NIR irradiation. Error bars represented the standard deviation. Asterisks (**) denoted significant differences (*p* < 0.01).

To explore the photothermal sensitivity of GPA, we evaluated the photothermal behavior of the GPA hydrogel under NIR irradiation in vitro. According to the heating curve obtained with NIR irradiation, the temperature of the GPA hydrogel increased from 20 to 43.5 °C in 5 min and reached 47.2 °C in 10 min under NIR irradiation (808 nm, 0.5 W cm^−2^), whereas the temperatures of PBS and GNR‐PEG hydrogel (GP) only increased from 20 to 29.1 and 45.5 °C, respectively (**Figure** **2**A). The increase in the temperature of the laser itself could not achieve a satisfactory photothermal effect. In contrast, the photothermal potency of the GPA hydrogel was 1.6‐fold higher than that of the NIR light itself. The photothermal efficiency of GPA hydrogel was calculated to be 26.5% (Figure S7, Supporting Information), and its photothermal activity remained stable even after five cycles of NIR irradiation (Figure 2B). These results further demonstrate the excellent photostability and efficient photothermal conversion effects of the GPA hydrogel.

To ensure the biosafety and feasibility of photothermal therapy for long‐term treatment of breast cancer recurrence and wound infection, the NIR‐induced hyperpyrexia around resected breast should maintained in a proper level, which could efficiently kill tumor cells and potential pathogenic bacteria without undesired damage for normal tissues. Although the photothermal efficiency of GPA hydrogel might not be preponderant to similar hydrogels, the quick response and excellent photothermal stability of GPA hydrogel was able to increase and maintain the temperature between 45 and 50 °C after NIR irradiation for 10 min (Figure S7, Supporting Information), which was suitable for tumor recurrence therapy and prevention of wound infection. Thus, the photothermal efficacy of GPA hydrogel could meet the requirements for synergistic treatment of breast cancer recurrence and wound infection.

Although no apparent difference was observed in the temperature increase between the GP and GPA hydrogels, the leakage of GNR from the GP hydrogel should be noticeable during long‐term implantation in vivo, which may lead to a sharp decrease in the photothermal effect. Therefore, we monitored the photothermal behavior in vivo using an infrared thermal camera under conditions involving NIR irradiation (808 nm, 0.5 W cm^−2^, 10 min) after the subcutaneous implantation of GPA hydrogel in mice (Figure 2C). After NIR irradiation for 5 min, the temperature of the GP and GPA hydrogels increased to 45.6 and 48.7 °C, respectively, whereas that of the PBS group remained unchanged before and after laser irradiation (Figure S8, Supporting Information). This result confirmed the remarkable photothermal activity of the GP and GPA hydrogels in vivo. Nevertheless, the enhanced temperature of the GP hydrogel reduced to 41.2 and 38.0 °C after 24 and 48 h of implantation, respectively, because GNR‐RGDY easily leaked from the GP hydrogel (Figure S9, Supporting Information). In contrast, the increasing temperature of the GPA hydrogel was maintained at 49.2 °C, suggesting that the hydrogel still possessed excellent photothermal properties even after 48 h of implantation. This effect was attributed to the tight fixation of GNR in the tight double network of the hydrogel, as previously mentioned.

Considering the superior photothermal effect of the GPA hydrogel, we explored the macroscopic phase transition of the gelation process under conditions involving laser irradiation of the mice skin to estimate the feasibility of NIR‐stimulated polymerization in vivo. As observed in Figure 2D, the precursor solution was completely converted to the sol state after NIR irradiation of the mice skin for 1 min, indicating that the GNR‐evocable photothermal effect resulted in thermally induced polymerization of PEGDA. This further confirmed that the injected GPA could be polymerized by NIR irradiation for the construction of a hydrogel in situ. Taken together, these results demonstrate the excellent photothermal conversion efficiency of the GPA hydrogel and highlight its application as a promising implant for local PTT in long‐term postoperative breast cancer treatment.

### 2.4 In Vitro Biocompatibility of Nanocomposite Hydrogels

To estimate biocompatibility in vitro, the cytotoxicity of PA, GPA, and GPA hydrogels against L929 cells under conditions involving laser irradiation was tested using the conventional CCK‐8 method. As shown in Figure 2E, the viability of L929 cells treated with PA, GPA, and GPA hydrogels and NIR irradiation was 95.7%, 87.8%, and 96.4%, respectively. As presented in Figure 2E, more than 90% cells in all groups were viable, corroborating that the hydrogel and nanoparticles used in this system exhibited good cytocompatibility.

Hemolysis of PA and GPA hydrogels was analyzed by incubating them with red blood cells (RBCs). As shown in Figure 2F, no apparent hemolytic activity was observed for PA, GPA, and GPA hydrogels from the calculated hemolysis ratios (all lower than 5.0%). These results further confirmed the improved biocompatibility of the GPA hydrogel in vitro.

### 2.5 In Vitro Antitumor Efficiency of Nanocomposite Hydrogels

Considering the excellent photothermal effects of GPA in vitro, 4T1 cells and breast tumor‐bearing mice were used to evaluate the synergistic radio‐photothermal antitumor efficiency in vitro. To explore the coordinating effects of brachytherapy and PTT, 4T1 cells were incubated with PA, GPA, and ^125^I‐GPA hydrogels with or without NIR irradiation (808 nm, 20 min, 0.5 W cm^−2^). The PBS group and PA hydrogel were used as the blank and negative control groups, respectively, to assess the tumor therapeutic potency of other treatment groups. The viability of 4T1 cells treated with ^125^I‐GPA and GPA with NIR irradiation was 68.2% ± 6.4% and 85.0% ± 3.6%, respectively, indicating that single RT or PTT demonstrated limited cytotoxic effects against cancer cells (Figure 2G). However, the viability of 4T1 cells sharply decreased to 15.8% ± 2.7% upon treatment with ^125^I‐GPA and laser irradiation, whereas no significant change was observed in the PA group before and after NIR irradiation. To further verify the antitumor capacity of the ^125^I‐GPA hydrogel on cells and visually observe the viable and dead cell distribution, the calcein‐AM live cell viability assay was performed for 4T1 cells treated with different samples using confocal laser scanning microscopy (CLSM). As depicted in Figure 2H, numerous dead cells were observed in the NIR‐irradiated ^125^I‐GPA hydrogel group, whereas few dead cells were observed in the photothermal group (PA hydrogel + NIR) and brachytherapy group (^125^I‐GPA hydrogel), indicating that massive numbers of cancer cells were killed through synergetic brachy‐photothermal therapy. In contrast, no apparent cell toxicity was observed in the NIR‐irradiated GNR or the laser‐irradiated group. These results suggest that the ^125^I‐GPA hydrogel enables superior brachy‐photothermal synergetic antitumor efficiency in vitro.

### 2.6 In Vivo Locoregional Inhibition of Breast Cancer Recurrence

**Figure 3:**
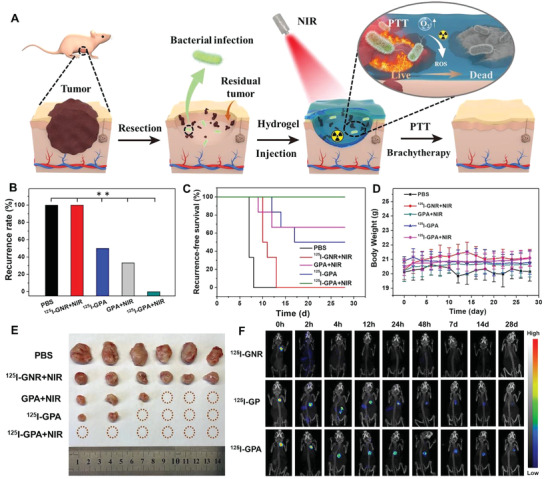
A) Schematic illustration of the therapeutic mechanism of the double‐network hydrogel (^125^I‐GPA) via PTT and brachytherapy for synergistic treatment of the postoperative breast cancer recurrence in infectious wounds. B) Breast cancer recurrence rate of PBS, ^125^I‐GNR, ^125^I‐GPA, and PA hydrogels with or without laser irradiation (*n* = 6). C) In vivo breast cancer locoregional recurrence curve of PBS, GNR, ^125^I‐GPA and PA hydrogels with or without laser irradiation (*n* = 6). D) Average body weights of the mice of different groups (*n* = 6). E) Photographs showing the gross tumor volume after treatment of different groups. F) SPECT images of the breast region before and after injection of ^125^I‐GNR, ^125^I‐GP, and ^125^I‐GPA hydrogels during 4 weeks implantation. Asterisks (**) denoted significant differences (*p* < 0.01).

Considering the excellent synergetic antitumor efficiency of brachytherapy and PTT in vitro, the ^125^I‐GPA hydrogel seems promising for the inhibition of breast cancer recurrence in vivo after resection of the primary tumor (**Figure** **3**A). In this study, mice were subcutaneously injected with PBS, GNR‐RGDY, PA, and ^125^I‐GPA precursor solutions in the resected breast cavity after tumor surgery and then irradiated for 20 min with a NIR laser to construct hydrogels. As shown in Figure 3B, different locoregional recurrence rates were examined after 4 weeks of treatment in 3 of 6 mice that were treated with ^125^I‐GPA hydrogel (Group 3, 50%) and 2 of 6 mice that were treated with GPA hydrogel with NIR irradiation (Group 2, 33.3%), and all of the 6 mice treated with the injection of PBS (Group 5) and GNR with laser irradiation (Group 4) exhibited recurrence (100%). Notably, the therapeutic group treated with ^125^I‐GPA hydrogel and NIR irradiation (Group 1) exhibited no locoregional tumor recurrence, suggesting that the synergetic photothermal and brachytherapy demonstrated better local inhibition of breast cancer recurrence (Figure 3C). In contrast, the inhibitory effects on tumor growth in the GNR group were limited owing to the quick elimination of nanoparticles from the tumor site. Therefore, the retention of radiolabeled GNR by in situ gelation of the hydrogel is essential to obtain long‐term photothermal effects and radioactivity.

Interestingly, the recurrence time of the photothermal treatment groups, including the GNR‐RGDY and GPA hydrogels with NIR irradiation, was earlier than that of the ^125^I‐GPA hydrogel (Figure 3C). A possible explanation is that the regional RT of ^125^I‐GPA hydrogel could continuously act during the whole growth stage of the tumor, especially during the radiation‐sensitive period. Although the tumor recurrence time in the brachytherapy group prolonged as compared to that in the PTT group, the recurrence rate of the brachytherapy group was higher than that of the PTT group. This observation is probably because PTT or brachytherapy alone was insufficient and ineffective in exerting satisfactory antitumor effects to prevent tumor recurrence. Hence, as demonstrated for the postoperative recurrence model of breast cancer, the in situ gelation of ^125^I‐GPA hydrogel enabled long‐term retention of radioactivity and excellent photothermal effects at the tumor surgical site and appeared to be a rather effective therapeutic approach for completely preventing postoperative tumor recurrence. In addition, an apparent decrease in the body weight was observed for the mice from the PBS control group, which was attributed to the detrimental effect of tumor progression on their physiological health (Figure 3D). In contrast, the body weight of mice from all treatment groups, including the thermal and brachytherapy groups, was maintained at a normal level, indicating that effective therapeutic intervention at the early stage could reduce tumor growth. Similar results were confirmed from the observation of the recrudescence of tumors in different groups after postoperative treatment for 28 days. As shown in Figure 3E, the tumor volume of the PBS group was remarkably larger than that of the other groups and was more than 10 times the volumes observed for GPA+NIR and ^125^I‐GPA groups.

Encouraged by the tight fixation of GNR in the DN hydrogel as mentioned above, we examined the in vivo isotope and CT imaging capability of hydrogels over 4 weeks. The ^125^I‐radiolabeled GPA (^125^I‐GPA) hydrogel was formed by subcutaneous injection into the postsurgical cavity of the breast after tumor resection under conditions involving NIR irradiation, and SPECT imaging was performed at different intervals. As shown in Figure 3F, the filling shape and implantation position of the ^125^I‐GPA hydrogel could be clearly recognized as compared with the surrounding tissue. The isotopic signal of ^125^I‐GNR diminished over time and completely vanished after 4 h because ^125^I‐GNR may be quickly eliminated in vivo. Conversely, the ^125^I‐GPA hydrogel was observed after 4 weeks, indicating its long‐term imaging ability in vivo. Notably, an irregular distribution of ^125^I‐GNR over 12 h was observed in the ^125^I‐PEG hydrogel owing to the explosive release of radiolabeled nanoparticles. These encouraging results revealed that the ^125^I‐GPA hydrogel could be tracked during the entire recurrence treatment of breast cancer employed in this study.

### 2.7 In Vivo Biocompatibility of Nanocomposite Hydrogels

**Figure 4:**
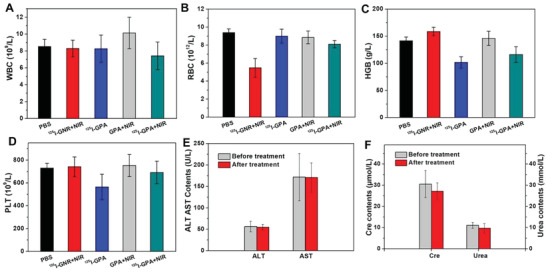
A–D) Routine blood test of different groups including white blood cell (WBC), red blood cell (RBC), hemoglobin (HGB), platelet (PLT) (*n* = 5). The function indices of liver and kidney including E) ALT, AST, F) creatinine and urea before and after treatment of ^125^I‐GPA hydrogel based on biochemical analysis (*n* = 5).

The in vivo biocompatibility of the nanocomposite hydrogels was investigated by hematoxylin and eosin (H&E) staining of the main organs, including the heart, liver, spleen, lungs, and kidneys, after treatment. No toxicity or inflammation was observed in any of the treatment groups compared to that in normal mice treated with PBS (Figure S10, Supporting Information). To further confirm the safety of the nanocomposite hydrogels, routine blood examinations involving white blood cell (WBC) and RBC counts and hemoglobin (HGB) levels were performed after 4 weeks of hydrogel implantation. As shown in **Figure** **4**A–D, no evident variations in the hematology markers were observed in any of the treatment groups except the ^125^I‐GNR group, which demonstrated the absence of inflammatory response or hematopoietic cell damage in the ^125^I‐GPA hydrogel. However, the decrease in RBC and WBC counts in the ^125^I‐GNR group further proved that the dissociative radiolabeled nanoparticles might induce radioactive damage to normal tissues. Furthermore, the functional indices of the liver and kidney were measured by biochemical analyses. As shown in Figure 4E,F, the functional indices before and after treatment with the ^125^I‐GPA hydrogel were within normal ranges compared to those in healthy mice. These results confirmed the better biosafety of the ^125^I‐GPA hydrogel.

### 2.8 In Vitro Anti‐Infective Property of Nanocomposite Hydrogels

**Figure 5:**
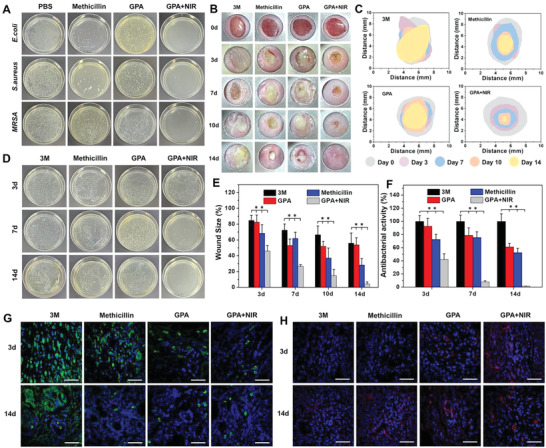
A) Images of bacteria clones on the agar plates after contacting with methicillin, GPA hydrogels with or without NIR irradiation against *E. coli*, *S. aureus*, and *MRSA*. B) Representative photographs of the infected wounds during 2 weeks treatment of different samples. C) Schematic images of wound infected area treated with different dressings. D) Photographs of bacteria clones on the agar plates of *MRSA* derived from wound tissues at 3, 7, and 14 days. E) Changes in wound size treated with various dressings (**p < 0.01, *n* = 5). F) Corresponding statistical data of colonies of *MRSA* derived from wound tissues for treatment of different dressings (**p < 0.01, *n* = 5). Immunofluorescence images of the infected tissues labeled with G) CD68 (green) and H) CD31 (red) treated with different dressings. Scale bar: 50 µm.

Surgical infection, a critical problem in the clinic, is often observed after surgery and is associated with more complex comorbidities. In particular, the emergence of antibiotic‐resistant pathogens remains a major threat to human health and survival.^[^ ^31^, ^32^ ^]^ Bacterial wound infection after surgery is one of the primary obstacles during postoperative treatment of cancer patients, and early intervention with antibacterial dressings could prevent contamination with pathogenic bacteria.^[^ ^33^, ^34^, ^35^ ^]^ Given the excellent photothermal performance of the GPA hydrogel, we initially evaluated the antibacterial capacity of the hydrogels against gram‐negative *Escherichia coli (E. coli)*, gram‐positive *Staphylococcus aureus (S. aureus)*, and methicillin‐resistant *Staphylococcus aureus (MRSA)*. After incubation with the GPA hydrogel under laser irradiation for 2 h, the majority of *S. aureus* (>90%) and *E. coli* (>90%) were inactivated, indicating the excellent antimicrobial activities against *S. aureus* and *E. coli*, while no antibacterial effect was observed for GPA hydrogel without laser irradiation compared to that in the PBS group (**Figure** **5**A). Meanwhile, the antibacterial efficacy of the GPA hydrogel with laser irradiation against resistant bacteria was also confirmed from the inhibition of *MRSA* and was higher than 95%. Although the antibiotic (methicillin) exhibited satisfactory inhibition of *S. aureus* (>90%) and *E. coli* (>90%), it had no potent effect against drug‐resistant bacteria (*MRSA*). Hence, this result suggests that PTT based on the GPA hydrogel was able to quickly and efficiently kill drug‐resistant bacteria.

To visually observe the antibacterial ability of the hydrogels, we evaluated their zones of inhibition against *E. coli*, *S. aureus*, and *MRSA*. In this experiment, larger diameters of the clear zone surrounding the disk revealed more effective antibacterial activity as compared to smaller diameters. As shown in Figure S11 (Supporting Information), the diameters of the zones of the GPA hydrogel with laser irradiation against *S. aureus* and *E. coli* were significantly larger than those of PBS and GPA hydrogel without laser irradiation, which achieved similar or even better antibacterial effects than methicillin. In particular, methicillin showed almost no inhibition of *MRSA*, implying its weak therapeutic effect in the treatment of drug‐resistant infections. These results further confirmed the excellent antibacterial effect of the GPA hydrogel with laser irradiation, rendering it a suitable candidate for anti‐infection therapy, especially for drug‐resistant pathogenic bacteria.

### 2.9 In Vivo Anti‐Infection Capacity of Nanocomposite Hydrogels

Considering that wound infection induced by drug‐resistant bacteria is one of the serious postoperative complications after tumor resection, we aimed to confirm the antibacterial efficacy of the GPA hydrogel in the presence of laser irradiation in an *MRSA*‐infected mouse model to mimic wound infection in resected breast cancer, as illustrated in Figure 5B–F. A full‐thickness skin incision was made on the backs of the mice in the presence of *MRSA*, and incisional wounds were treated with GPA hydrogel with or without laser irradiation. Infected wounds treated with commercial 3M dressing and methicillin were used as negative and positive control, respectively, to evaluate the anti‐infectious effect of the NIR‐assisted GPA hydrogel.

At the beginning of the treatment, the incisions treated with the GPA hydrogel under laser irradiation began to close and scab, indicating that the pathogenic bacteria were eliminated and the wound started to heal, whereas other groups exhibited more severe infection and larger infected areas (Figure 5B). Although the size of the incision area decreased in the 3M dressing group, the infection was not inhibited and led to severe inflammation. After treatment for 10 days, the incisions in the mice treated with NIR‐assisted GPA hydrogel exhibited a well‐healed wound, but wound infection persisted in other groups. Despite the alleviation of wound size and infection in the methicillin group than that in the 3M group, the inflammation and damage were more serious than those in the NIR‐assisted GPA hydrogel. This observation indicated the weak antibacterial activity of traditional antibiotics in this drug‐resistant model. Furthermore, less hair was observed near the wound in 3M and methicillin groups, indicating skin damage caused by bacterial infection. Notably, the wound infected by drug‐resistant bacteria was almost completely healed without scars after 2 weeks in the NIR‐assisted GPA hydrogel group, while the other treatment groups still exhibited unhealed incision and local infection. The excellent antibacterial activity might be attributed to the fact that the tightly fixed GNRs in the DN PA hydrogel were able to maintain better photothermal efficiency in vivo. In addition, the desired functionality of alginate and the enhanced blood circulation mediated by PTT were conducive to wound healing.

To further evaluate the wound healing performance of the hydrogels with NIR irradiation, we examined the variations in the infected areas in all groups during 14 days of treatment. As depicted in Figure 5C, wounds treated with hydrogels under NIR irradiation showed significant contraction on day 7, while no apparent contraction was observed in other groups. Thus, the wound infection was relieved after PTT of the GPA hydrogel. On day 14, the infected wound size in mice treated with 3M dressing was not suppressed and was in the same range as that of the initial wound (Figure 5E), revealing that the traditional dressing for wound infection was ineffective. A similar result was observed in the methicillin group, demonstrating that the conventional antibiotic showed unfavorable response against drug‐resistant infections.

To confirm the in vivo anti‐infection effect of the NIR‐irradiated hydrogel, the residual bacteria were collected from the wounded tissue after treatment and cultured overnight on Luria Bertani (LB) agar plates for quantitative determination. On day 3, the number of bacteria in the NIR‐irradiated GPA hydrogel was found to be significantly lower than that in the 3M dressing group (Figure 5D), demonstrating effective anti‐infectious effects in vivo. On day 7, no apparent change was observed in the bacterial count of 3M, methicillin, and GPA hydrogel groups; in contrast, the antibacterial efficiency of the NIR‐irradiated GPA hydrogel group increased to 91.8% (Figure 5F). On day 14, almost no bacterial growth was observed in the NIR‐irradiated GPA hydrogel group, whereas 3M, methicillin, and GPA hydrogel groups exhibited high bacterial growth. These results indicated that PTT based on the GPA hydrogel was able to effectively inhibit drug‐resistant bacterial infection in vivo, thereby promoting wound healing in the case of bacterial infection.

To estimate infected wound healing, the wounded tissues were subjected to H&E staining on days 3, 7, and 14. As displayed in Figure S12 (Supporting Information), the wound treated with the GPA hydrogel under NIR irradiation showed better closure of incision than the wound from the control group on day 7. This observation is consistent with the results discussed in Figure 5B. Moreover, less inflammatory infiltration and more collagen deposition were observed in the NIR‐irradiated hydrogel group than in the 3M dressing group, indicating better post‐wound closure care of the GPA hydrogel under NIR irradiation. After treatment for 2 weeks, more granulated tissues with denser structures and skin appendages, such as hair follicles, were observed in the NIR‐irradiated GPA hydrogel group than those in the 3M and methicillin groups. Moreover, the NIR‐irradiated GPA hydrogel group showed almost complete regeneration of the dermal tissue with skin and enhanced angiogenesis as compared to the 3M dressing group. These results demonstrate that inhibition of bacterial infection may accelerate the wound healing process and regeneration of the skin tissue. Taken together, these intriguing results corroborate the promising application of the GPA hydrogel as a soft filler to inhibit wound infection and inflammation.

### 2.10 In Vivo Infection‐Induced Wound Healing

Macrophages are important for wound healing, particularly for the relief of inflammation. CD68, which belongs to the lysosomal glycoprotein family, is specifically expressed by tissue macrophages.^[^ ^36^ ^]^ Therefore, immunofluorescence staining of CD68 in various treatment groups was performed to evaluate the effect of the inflammatory response. As shown in Figure 5G, the expression of CD68 in the NIR‐irradiated GPA hydrogel group was significantly lower than that in the 3M and methicillin‐treated groups on day 3. Furthermore, a lower inflammatory response at the skin wound was observed for the NIR‐irradiated GPA hydrogel group than for the other three groups, demonstrating the excellent anti‐infectious capacity of the NIR‐assisted hydrogel. Although the expression of CD68 reduced in the 3M dressing group on day 14, the inflammatory response in the wounded tissue treated with 3M dressing was still far more serious than that in other treatment groups, indicating that early interventions targeting bacterial infection, such as antibacterial therapy, may decrease the local inflammatory response. These results demonstrated that the nanocomposite hydrogel with NIR‐activated antimicrobial activity was capable of reducing the inflammatory response during drug‐resistant infection and thereby accelerating wound healing.

The presence of endothelial cell tissue and angiogenesis was demonstrated by immunofluorescence of CD31, which is a transmembrane protein expressed in early angiogenesis that acts as a signaling molecule to regulate angiogenesis.^[^ ^37^ ^]^ On day 3, a high expression of CD31 was observed by immunohistochemical staining in GPA+NIR group (Figure 5H), whereas no expression of CD31 was observed in other treatment groups. After treatment for 2 weeks, the NIR‐irradiated GPA hydrogel showed the highest expression of CD31 as compared to the other treatment groups, indicating that the NIR‐irradiated GPA hydrogel could upregulate the expression of CD31, and thereby, promote wound healing.

## 3 Conclusion

In summary, we developed a tumor‐targeted nanocomposite double‐network (NDN) hydrogel obtained by NIR‐induced polymerization of PEGDA and endogenous Ca^2+^‐crosslinked ALG with the addition of radioisotope‐labeled ^125^I‐GNR‐RGDY. The NDN hydrogel exhibited excellent photothermal effects and radiolabel stability and demonstrated high therapeutic efficacy with synergistic PTT and brachytherapy. On account of these intriguing features, the nanocomposite hydrogel was formed by in situ injections of precursor solutions into the cavity of postoperative cancerous breast tissue through polymerization of PEGDA induced by NIR light illumination, followed by crosslinking between endogenous Ca^2+^ and ALG to construct the DN hydrogel. Upon NIR irradiation, the triggered hyperpyrexia induced by the built‐in photothermal effect of ^125^I‐GNR‐RGDY inhibited self‐repair of the radiation‐induced DNA damage and promoted blood circulation to alleviate the hypoxic environment, thereby improving the antitumor efficiency of brachytherapy. Furthermore, photothermal ablation could simultaneously eliminate potential pathogenic bacteria to prevent postoperative wound infection. During the 4‐week treatment with the NDN hydrogel, synergism between the photothermal effect and continuous brachytherapy efficiently prevented breast cancer recurrence and wound infection. Notably, the embedded ^125^I‐GNR‐RGDY endowed the NDN hydrogel with long‐term isotope‐imaging properties. The novel strategy reported here points out a new direction for precise postoperative treatment of breast cancer recurrence and wound infection with a single injectable nanocomposite DN hydrogel exploiting the combination of PTT, brachytherapy, and self‐imaging.

## 4 Experimental Section

### Materials

Polyethylene glycol acrylate (PEGDA, *M*_n_ 700), sodium alginate (ALG), 1‐ethyl‐3‐(3‐dimethylaminopropyl) carbodiimide hydrochloride (EDC), *N*‐hydroxysuccinimide (NHS), and 2,2′‐azobis [2‐(2‐imidazolin‐2‐yl)propane] dihydrochloride (AIPH) were purchased from Sigma‐Aldrich. PEG modified Au nanorods (GNR) was supplied by XFNANO (Nanjing) Co. Ltd. RGDY peptide was provided by GL Biochem (Shanghai) Co. Ltd. Iodine‐125 (^125^I) radionuclide (500 mCi mL^−1^ in 0.1 m NaOH solution) was obtained from PerkinEmer (USA).

### Synthesis of ^125^I‐GNR‐RGDY

RGDY peptide (10 mg) was first was dissolved in PEG modified GNR solution (10 mL, 1 mg mL^−1^) and then NHS (1 × 10^−3^ m, 23 mg) as well as EDC (1 × 10^−3^ m, 38 mg) were added to the solution. The mixture was stirred overnight at room temperature, followed by centrifugation/dispersion twice with Mili‐Q water for 2 min and then lyophilized, to obtain the final GNR‐RGDY. The variation of absorption property was estimated by UV–vis spectrum based on the intensity of GNR's characteristic absorption peak (520 and 820 nm).

The ^125^I radiolabeling process was according to a previously reported method.^[^ ^30^, ^38^, ^39^ ^]^ Briefly, the GNR‐RGDY was dissolved in Mili‐Q water and then chloramine‐T (1 µmol) in PB (pH 7.4, 10 × 10^−3^ m, 100 µL) as well as Na^125^I (2 mCi) in PB (1 mL) was added, respectively. The mixture was shaken at room temperature for 5 min, followed by adding Na_2_S_2_O_5_ (2 µmol) in PB (100 µL) to quench the reaction. Then, the reaction mixture was centrifugated/dispersed twice with Mili‐Q water and lyophilized to obtain the final product ^125^I‐GNR‐RGDY. Notably, the ^125^I‐GNR‐RGDY was only used in cell and animal experiments due to restriction of isotope application.

### Preparation of GPA Hydrogel

ALG and PEGDA (mass ratio 1:9) were first dissolved in GNR‐RGDY solution at a 20 wt% monomer concentration, and then 1 wt% monomer content of thermalinitiator AIPH was added into the solution and stirred thoroughly until completely dissolved. Subsequently, the mixture was irradiated by near‐infrared light (NIR, 0.5 W cm^−2^) for 20 min to induce the polymerization of PEGDA. The obtained hydrogels were then immerse into Ca^2+^ solution (10 × 10^−3^ m) for 24 h to activate the double network duo to the crosslink reaction between ALG and Ca^2+^. The double‐network nanocomposite hydrogel was named as GPA hydrogel. Similarly, the ^125^I‐radiolabeled GPA hydrogel was synthesized by dissolving the monomers into the aqueous solution of ^125^I‐GNR‐RGDY.

### In Vivo SPECT Imaging

Mice were randomly divided into three groups (three mice per group) and threated with the ^125^I‐GNR, ^125^I‐GP, and ^125^I‐GPA, respectively. Same radiation dose (100 µCi per mouse) of each group was used in this experiment. The in vivo SPECT imaging was collected at 0, 2, 4, 12, 24, 48 h, 7, 14, and 28 d via nanoscan SPECT/CT (Mediso) to investigate the location of the hydrogels.

### In Vivo Inhibition of Locoregional Breast Cancer Recurrence

All animal procedures were performed in accordance with the Guidelines for the Care and Use of Laboratory Animals of Peking Union Medical College and experiments were approved by the Animal Experiments and Ethics Review Committee of the Institute of Radiation Medicine, Chinese Academy of Medical Sciences (No. IRM‐DWL‐2021043). BALB/c mice (*n* = 6) were supplied by Beijing Vital River Laboratory Animal Technology Co., Ltd., China (female, 4–6 weeks old). Then, 4T1 cells (10^6^ cells in 100 µL PBS) per mouse were injected into the right thoracic mammary fat pad to produce orthotopic primary tumors. Tumor were resected when it reached 200 mm^3^ after ≈2 weeks. To ensure a high rate of recurrence in control group, the covering and surrounding kin of the tumors was preserved. Group 1: ^125^I‐GPA hydrogel implant with laser irradiation; Group 2: nonradioactive GPA hydrogel implant with laser irradiation; Group 3: ^125^I‐GPA hydrogel implant without laser irradiation. The same dosage of ^125^I‐GNR were injected subcutaneously (Group 4) into the mice's breast with laser irradiation as the positive control, respectively. The mice treated with PBS and without laser irradiation served as the negative group (Group 5). All radioactive treatment groups received a radiation dose of 100 *μ*Ci.

### In Vivo Antibacterial Efficacy against Localized Bacterial Infection

The bacterial drug‐resistance infection wound models were established on BALB/c mice under anesthesia with isoflurane. After local cleaning and disinfection, a 7 mm diameter wound on mice back was made by sterilized scalpel. The wounds were then washed with PBS and dropped with 20 mL of MRSA solution (10^8^ CFU mL^−1^). The mice (*n* = 5) were randomly divided into four treated groups: 1) PBS; 2) methicillin; 3) GPA hydrogel; 4) GPA hydrogel with NIR irradiation. The PBS and methicillin was used as negative and positive control group, respectively. For groups 4, the wounds were treated with 808 nm laser irradiation (0.5 W cm^−2^, 5 min). The wound size and infection was observed every 2 days. During 2 weeks antibacterial treatment, the wound tissues were excised from mice for H&E staining to evaluation the wound healing.

### Statistical Analysis

Both in vitro and in vivo experiments were analyzed by the one‐way ANOVA with Tukey’ post hoc test and expressed as means ± standard deviations (SD). The student's *t*‐test was utilized to determine whether data groups differed significantly from each other. Statistical significance was defined as having *p < 0.05, **p < 0.01. SPSS 20.0 was used for statistical analysis of data.

## Conflict of Interest

The authors declare no conflict of interest.

## Supporting information

Supporting Information

## Data Availability

Research data are not shared.
