# Supplementary material for: Tumor‐Targeted Injectable Double‐Network Hydrogel for Prevention of Breast Cancer Recurrence and Wound Infection via Synergistic Photothermal and Brachytherapy
Source: Adv Sci (Weinh). 2022 Jun 25;9(24):2200681. doi: 10.1002/advs.202200681 (PMC9403641; doi:10.1002/advs.202200681)
Supplement: Supplementary file 1 — Supporting Information [file ADVS-9-2200681-s001.pdf]

ADVANCED SCIENCE
Open Access

Supporting Information

for *Adv. Sci.*, DOI 10.1002/advs.202200681

Tumor-Targeted Injectable Double-Network Hydrogel for Prevention of Breast Cancer Recurrence and Wound Infection via Synergistic Photothermal and Brachytherapy

Yuanhao Wu, Yuan Yao, Jiamin Zhang, Han Gui, Jinjian Liu and Jianfeng Liu**

Supporting information

Tumor-Targeted Injectable Double-Network Hydrogel for Prevention of Breast Cancer Recurrence and Wound Infection via Synergistic Photothermal and Brachytherapy

Yuanhao Wu, Yuan Yao, Jiamin Zhang, Han Gui, Jinjian Liu and Jianfeng Liu**

Y. Wu, J. Zhang, H. Gui, J. Liu, Prof. J. Liu

Key Laboratory of Radiopharmacokinetics for Innovative Drugs, Chinese Academy of Medical Sciences, and Institute of Radiation Medicine, Chinese Academy of Medical Sciences & Peking Union Medical College, Tianjin, 300192, China

E-mail: liujinjian@irm-cams.ac.cn, liujianfeng@irm-cams.ac.cn

Y. Yao

Lab of Functional and Biomedical Nanomaterials, College of Materials Science and Engineering, Qingdao University of Science and Technology, Qingdao 266042, China

Keywords: double-network hydrogel, breast cancer, wound infection, brachytherapy, photothermal therapy

Experimental Section

Characterization: The morphology of the GNR was observed by transmission electron microscopy (TEM) (JEM-2100, Japan). Scanning electron microscopy (SEM) was conducted at an accelerate voltage of 5 kV using a FEI Quanta S-4800 microscope (Japan). The hydrogel samples were flash frozen in liquid nitrogen for 5 min and immediately lyophilized for 2 days to remove water. Au coating of samples for imaging was carried out by sputtering for 45 s. UV–Vis spectrophotometry measurements were performed on TU-1810 UV–Vis spectrophotometer with a wavelength range from 400 to 1000 nm. *In vivo* isotope imaging was carried out by using SPECT-CT system (Triumph X-SPECT2h/X-OCT).

Cytotoxicity Assay: CCK-8 was used to evaluate the cell cytotoxicity of the nanoparticles and polymers used in this experiment. Before detection, the sample disks (5 mm in diameter) were cut from hydrogel films, immersed in 75% ethanol for 2 h for sterilization and then washed with PBS overnight. Subsequently, the PA and GPA samples were placed in a 96-well plate, and fixed on the bottom of the wells. Fibroblasts L929 suspension (100 μL) was added into 96-well microplates, with 5000 cells immersed in the complete growth medium per well, cultivated in a humidified 5% CO_2 atmosphere at 37 °C for 24 h to allow cells to attach. Then, CCK-8 solution was added to 96-well plates at 10 μL per well and incubated for 3 h. The resulting solutions were analysed at 480 nm on a plate reader (BIO-TEK instruments Inc EL311S, America). This process was repeated for 8 times in parallel. The results were expressed as the relative cell viability (%) with respect to blank group only with culture medium. The cell viability in each well was calculated from the obtained values as a percentage of control wells. The results were presented as a mean and standard deviation obtained from each samples.

In Vitro Antibacterial Evaluation of the Hydrogels: Escherichia coli (E. coil), Staphylococcus aureus (S. aureus) and Methicillin-resistant Staphylococcus aureus (MRSA) were employed to test the antibacterial activity of the hydrogel. In brief,

bacteria were divided into four groups with different treatments: (1) PBS; (2) methicillin; (3) GPA hydrogel; (4) GPA hydrogel with NIR irradiation. The prepared hydrogel samples were added into 48-well plate and then 10 μL bacterial suspension (in PBS, 10^6 CFU/mL) was added onto the surface of hydrogel disks. Next, this 48-well plate was put into incubator at 37 °C for 2 h in a relative humidified atmosphere. At the end of that time, 1 mL of sterilized PBS was added to each well to re-suspend any bacterial survivors. As the control, 10 μL of bacterial suspension in sterilized PBS (10^6 CFU/mL) was added in 1 mL PBS to obtain a homogeneous solution. After incubation for 18-24 h at 37 °C, the colony-forming units (CFU) on the Petri dish were counted. Tests were repeated three times for each group and the killing ratios of bacteria by hydrogels were measured.

Hemolysis Assay: Fresh blood was obtained from SD rats, whose erythrocytes were separated by centrifugation at 3000 rpm for 10 minutes, washed three times with saline solution, and finally diluted with erythrocyte stock (100 μL, 5%). The samples of PA and GPA was added to the solution, respectively, and incubated for 1 h at 37 °C. Then the samples were centrifuged at 1000 rpm for 5 minutes and the supernatants were collected for further detection. Hemolytic activity was determined by OD_{545nm} using a multifunctional microplate reader. Red blood cells (RBC) in saline solution was a negative control and deionized water was a positive control. The hemolysis percentage (Hemolysis %) was calculated from the following equation:

$$\text{Hemolysis\%} = [(A_S - A_-) / (A_+ - A_-)] \times 100\% \qquad (2)$$

where A_S is the absorbance of the samples, A_- is the absorbance of the negative control and A_+ is the absorbance of the positive control.

Biochemical Analysis: The mice's serum samples of treatment group and control group (normal rat) were analyzed for a wide range of general biochemical parameters such as alanine aminotransferase (ALT), aspartate aminotransferase (AST), urea and creatinine. These parameters were measured with standard automated biochemical analyzers.

Hematology Analysis: The hematology analysis was carried out to evaluate the in vivo biocompatibility of the hydrogels after tumor therapy. Briefly, 6–8 weeks old healthy BALB/c mice were randomly divided into five groups (five mice per group): the PBS group, free ^{125}I-GNR+NIR group, ^{125}I-PA group, PA+NIR group and ^{125}I-PA+NIR group. After 28 days of cancer recurrence treatment, blood samples were obtained from the eyeball of the mice in each group and then treated with anticoagulants. An automatic bloodanalyzer (Nihon Kohden, MEK-7222K) was used for the hematological examination, including the analysis of RBC, WBC, HGB and PLT.

Pathology Analysis: On the 28th day after treatment, the mice were euthanized, and the representative organs including heart, liver, spleen, lung, and kidney were excised, fixed with a 4% paraformaldehyde solution overnight, embedded in paraffin, sliced into 8 μm thick sections, and stained with H&E. Ultimately, the slices were observed and photographed using an optical microscope (Leica, DMI 3000 B) for the pathology analysis to evaluate the biosafety of the hydrogel during the treatment.

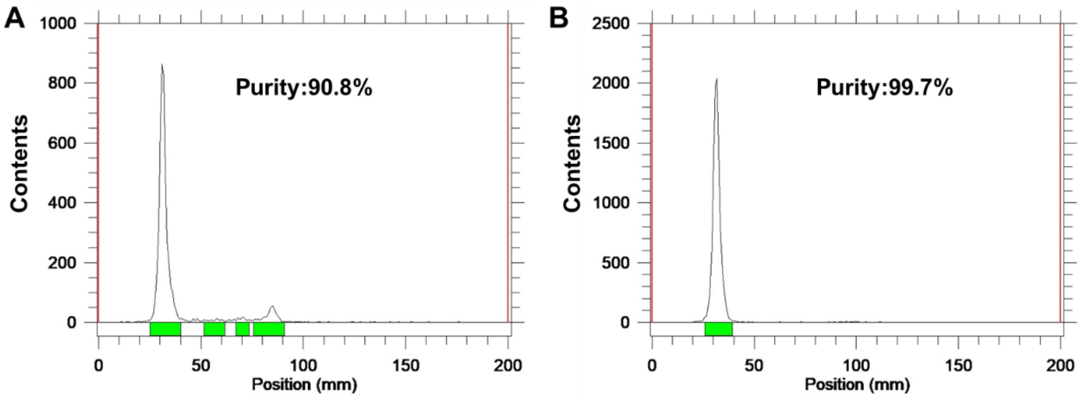

Figure S1. The radiolabeling rate A) and radiochemical purity B) of the ^{125}I-RGDY-GNR determined by a radioactive TLC scanner.

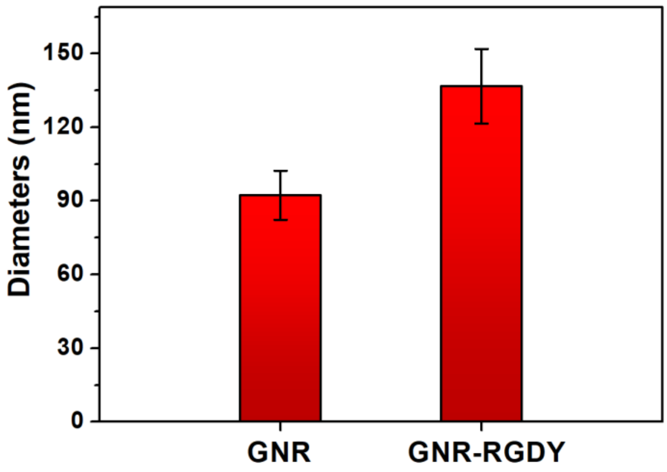

Figure S2. Hydrodynamic diameters of GNR and GNR-RGDY.

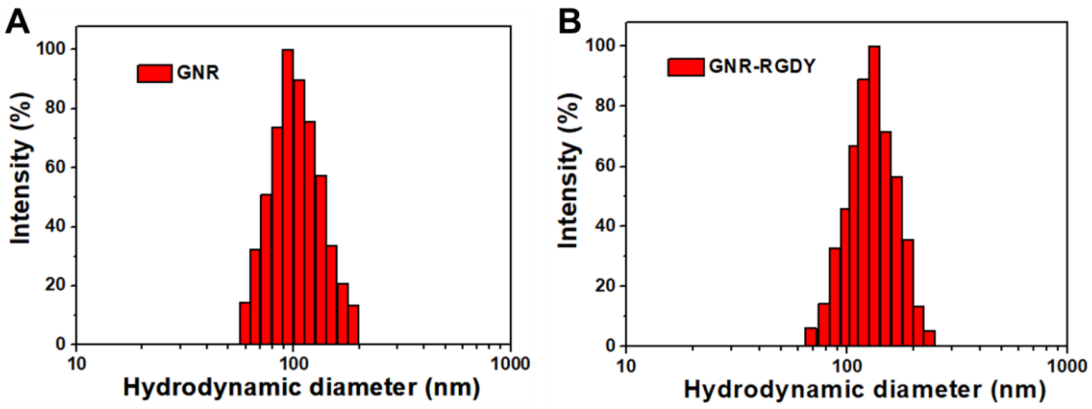

Figure S3. Hydrodynamic diameter distributions of GNR and GNR-RGDY.

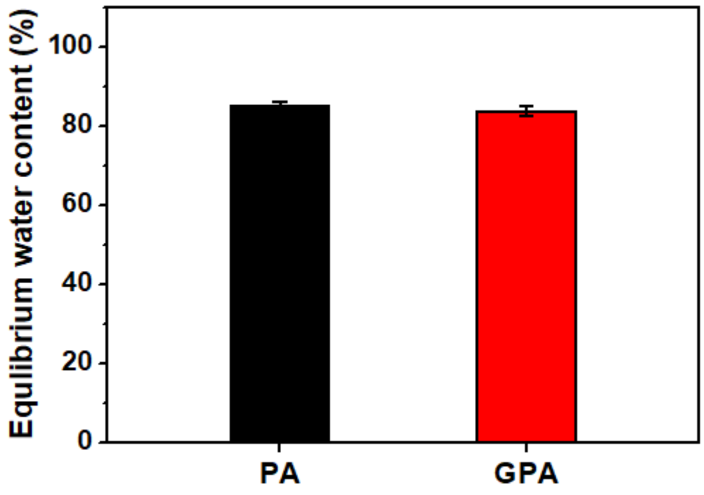

Figure S4. Equlibrium water content of PA and GPA hydrogel.

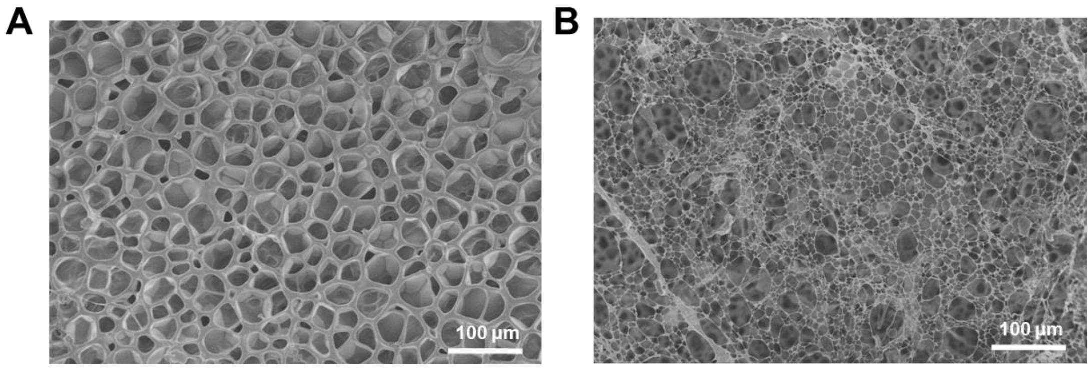

Figure S5. SEM images of PEG and PA hydrogels.

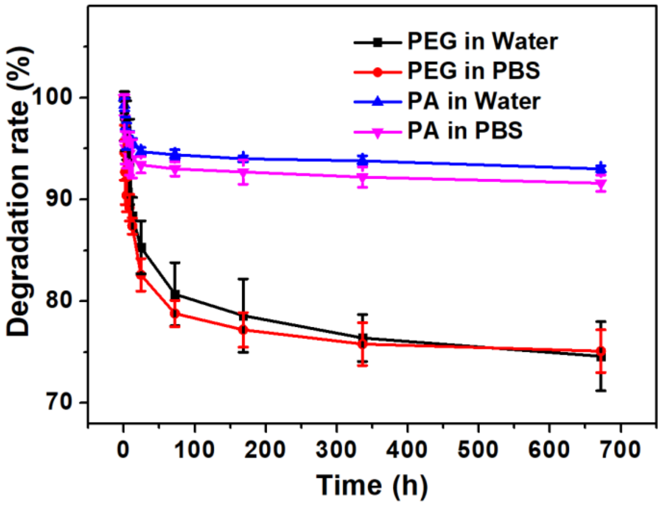

Figure S6. Degradation rate of different hydrogels in water and PBS during 4 weeks.

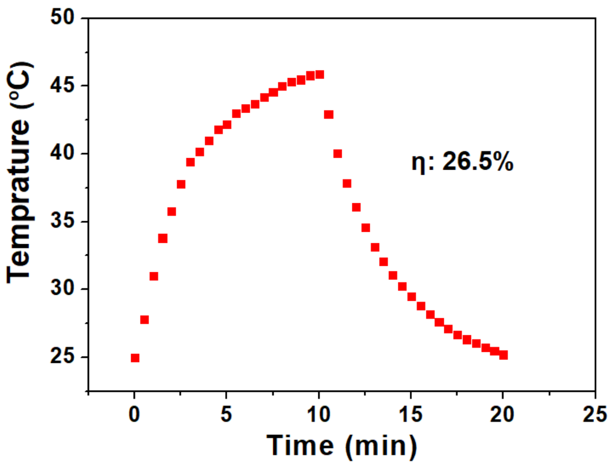

Figure S7. Photothermal conversion efficiency of GPA hydrogel.

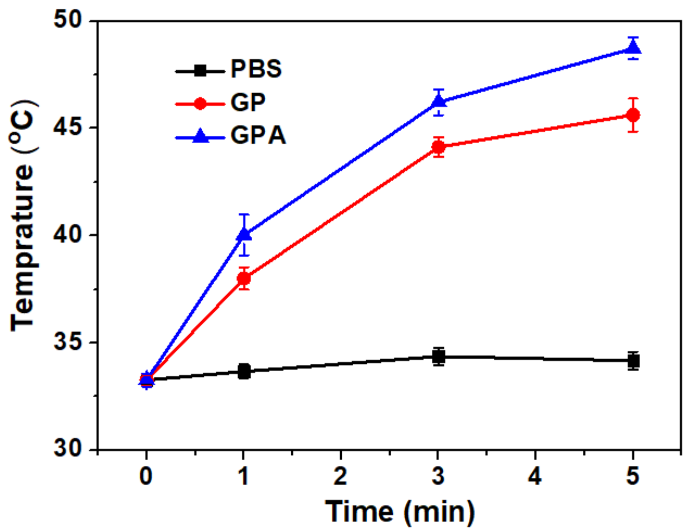

Figure S8. *In vivo* photothermal activity of PBS, PA and GPA hydrogels under NIR irradiation in 5 min.

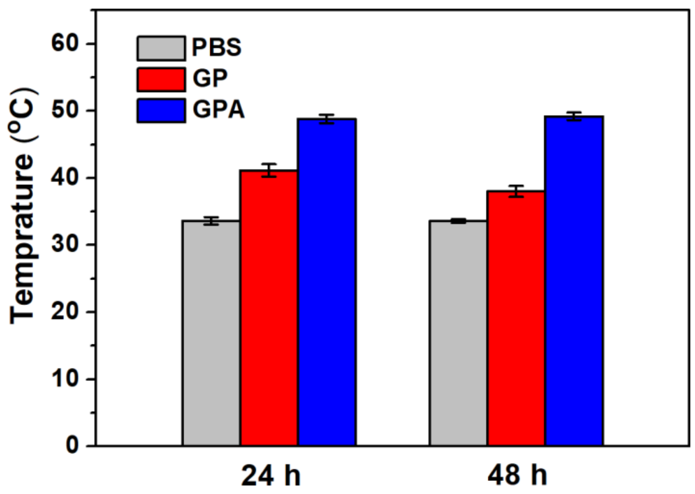

Figure S9. *In vivo* photothermal activity of PBS, PA and GPA hydrogels under NIR irradiation after implantation for 24 h and 48 h.

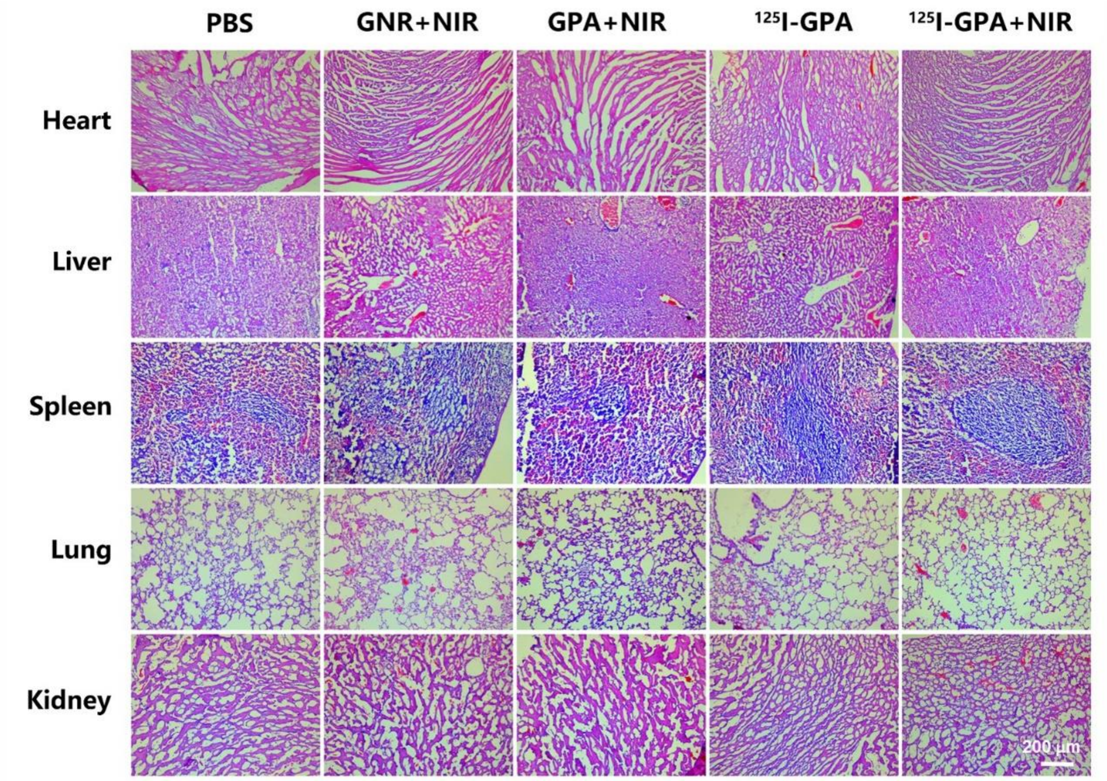

Figure S10. H&E stained images of the heart, liver, spleen, lung, and kidney treated with PBS, GNR + NIR, ^{125}I-PA hydrogel and ^{125}I-PA hydrogel + NIR.

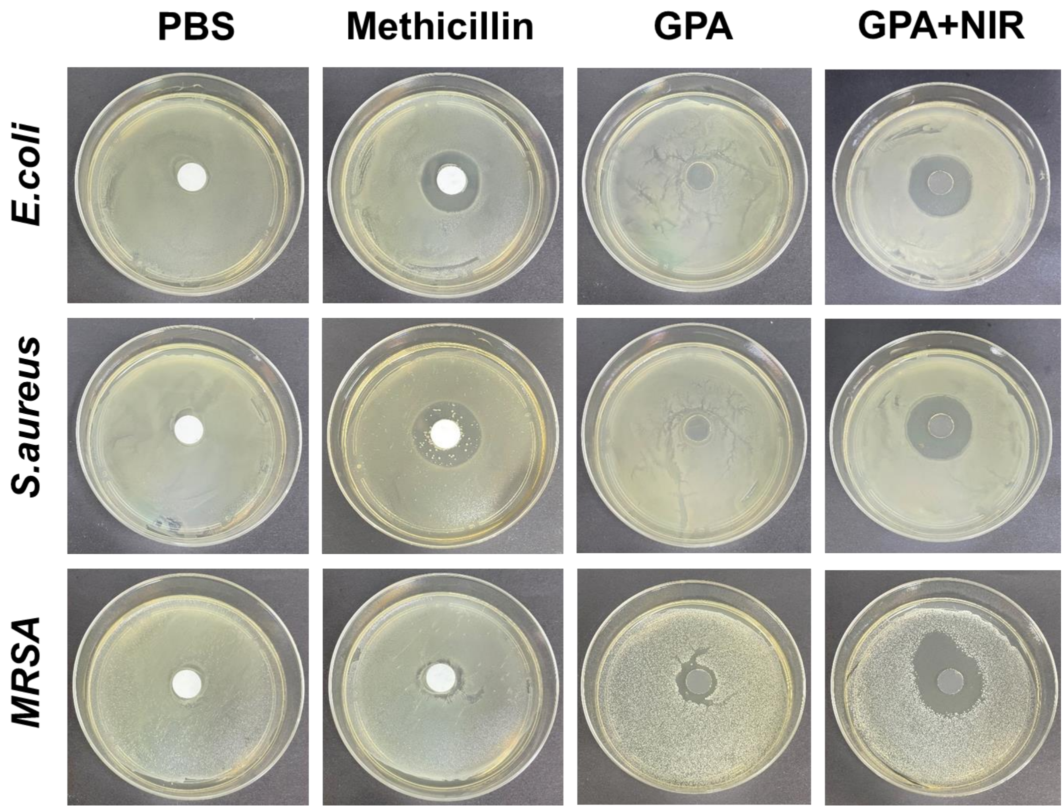

Figure S11. Inhibition zones of PBS, methicillin, GPA hydrogel with or without NIR irradiation against *E. coli, S. aureus and MRSA.*

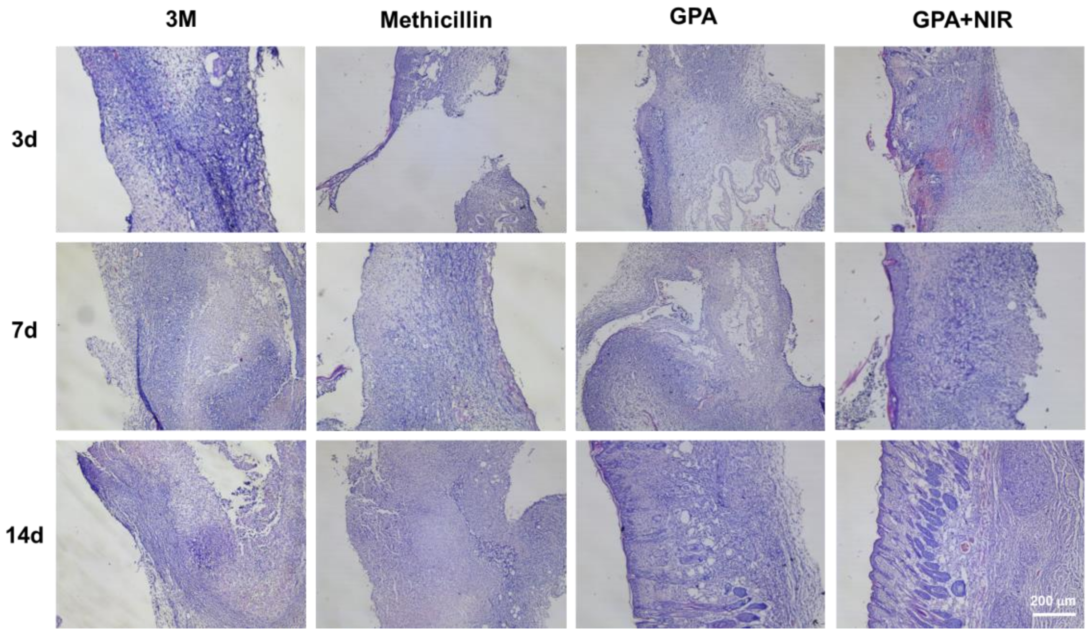

Figure S12. H&E-staining results of healed wound tissues of different treatment groups at determined times.
